# Recent progress on the microbial mitigation of heavy metal stress in soybean: overview and implications

**DOI:** 10.3389/fpls.2023.1188856

**Published:** 2023-06-12

**Authors:** Shifa Shaffique, Saddam Hussain, Sang-Mo Kang, Muhammad Imran, Eun-Hae Kwon, Muhammad Aaqil Khan, In-Jung Lee

**Affiliations:** ^1^ Department of Applied Biosciences, Kyungpook National University, Daegu, Republic of Korea; ^2^ Department of Agronomy, The University of Agriculture Faisalabad, Faisalabad, Pakistan; ^3^ National Institute of Agriculture Science, Rural Development Administration, Biosafety Division, Jeonju, Republic of Korea; ^4^ Department of Chemical and Life Sciences, Qurtuba University of Science and Information Technology, Peshawar, Pakistan

**Keywords:** heavy metal, soybean, microbes, mitigate, ROS

## Abstract

Plants are adapted to defend themselves through programming, reprogramming, and stress tolerance against numerous environmental stresses, including heavy metal toxicity. Heavy metal stress is a kind of abiotic stress that continuously reduces various crops’ productivity, including soybeans. Beneficial microbes play an essential role in improving plant productivity as well as mitigating abiotic stress. The simultaneous effect of abiotic stress from heavy metals on soybeans is rarely explored. Moreover, reducing metal contamination in soybean seeds through a sustainable approach is extremely needed. The present article describes the initiation of heavy metal tolerance mediated by plant inoculation with endophytes and plant growth-promoting rhizobacteria, the identification of plant transduction pathways via sensing annotation, and contemporary changes from molecular to genomics. The results suggest that the inoculation of beneficial microbes plays a significant role in rescuing soybeans under heavy metal stress. They create a dynamic, complex interaction with plants via a cascade called plant–microbial interaction. It enhances stress metal tolerance via the production of phytohormones, gene expression, and secondary metabolites. Overall, microbial inoculation is essential in mediating plant protection responses to heavy metal stress produced by a fluctuating climate.

## Graphical Abstract

A schematic representation of the mitigation of heavy metal stress via plant–microbial interaction.

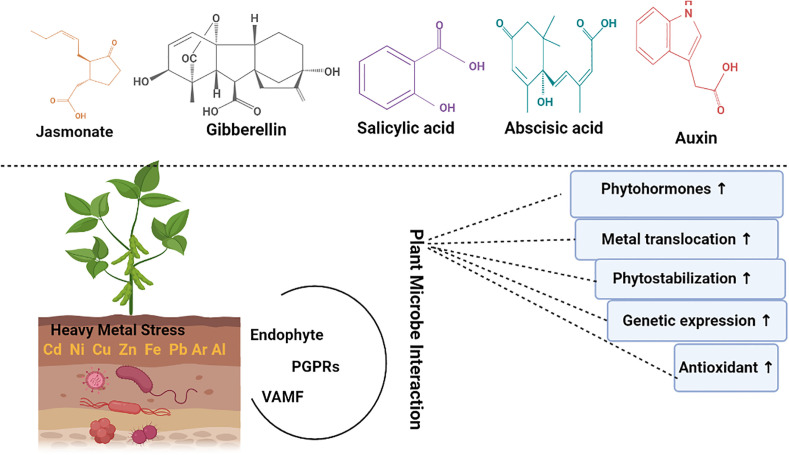

## Introduction

Environmental stress is a persistent threat to agriculture production. In the current era, pollution of the environment with heavy metals is increasing rapidly. The accumulation of heavy metals in the environment is a serious concern globally for scientists because they exert long-lasting toxic effects on ecosystems (De Storme and Geelen, 2014; Pessarakli et al., 2015). Heavy metal stress is abiotic, which is alarming for the ecological system and disturbs the biotic and abiotic components of ecological systems. As such, it degrades soil texture, pH, and mineral content. In addition, it causes heavy metal toxicity in plants, reducing crop productivity. The agriculture industry is facing pressure to meet the zero-hunger population, and the human population is increasing and is estimated to reach 10 billion people worldwide by 2050. In addition, agronomy has to face abiotic stresses, such as heavy metal stress, the most prevalent stress globally (Conforti, 2011; Alexandratos and Bruinsma, 2012; Stout, 2012). Higher plants are sessile in nature, so they have to face constant environmental stress. Heavy metal toxicity is the leading cause of reduced crop productivity among different abiotic stresses. Heavy metal stress can be defined as a metal with a specific density of more than 5 g/cm^3^ (Maksymiec, 2007; Bilal et al., 2020). The most distinguishable characteristics of heavy metals are their high density and greater atomic number of more than 20. Among 90 metals, only 17 are labeled as beneficial, while 53 others are designated as heavy metal stressors (Polle and Schützendübel, 2003; Tuteja et al., 2011). Among beneficial metals, iron, zinc, and copper are required only in trace amounts for plant health, but in excess amounts, they cause heavy metal stress (Mani and Sankaranarayanan, 2018; Emamverdian et al., 2020). It is estimated that more than a million hectares of cultivable land are contaminated with heavy metals in China, which is a total of 20% of total land biomass (Yadav et al., 2020).

**Figure 1:**
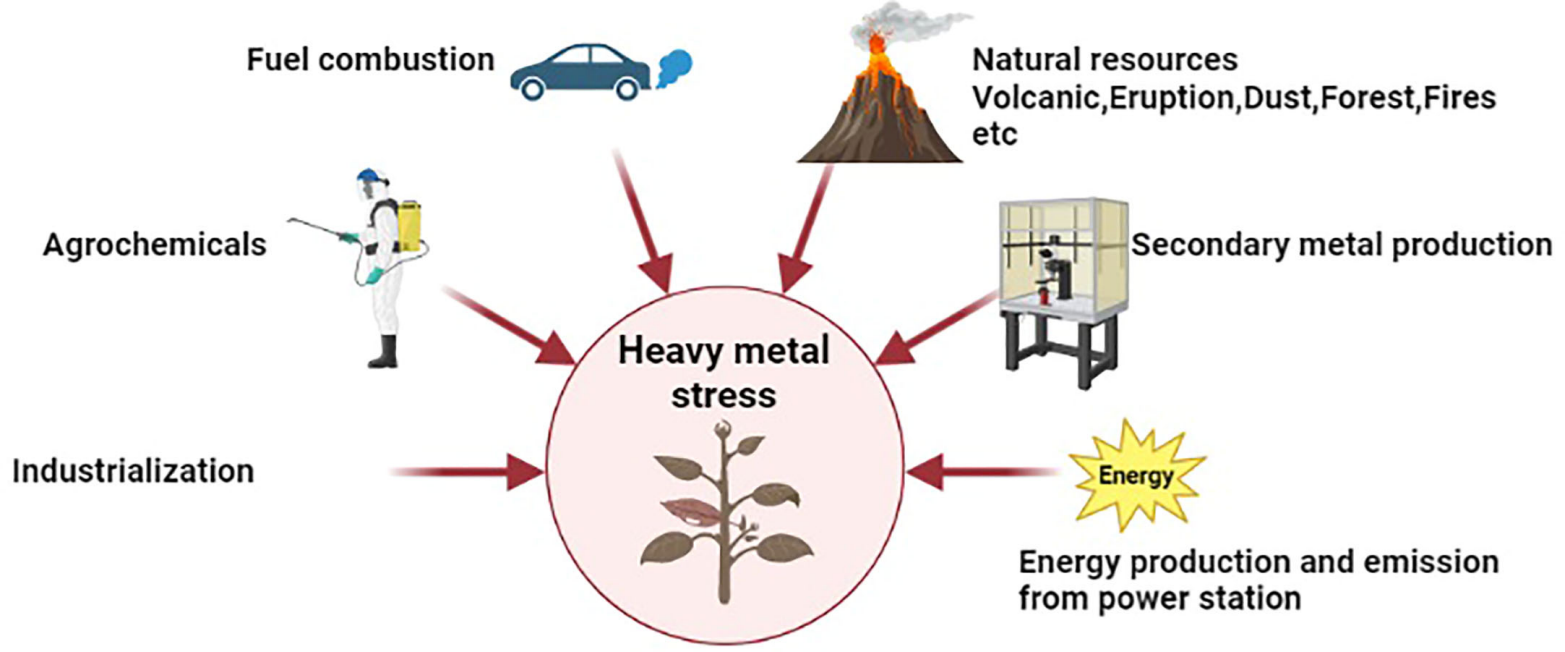
Sources of heavy metal stress on plant performances.

Agrochemical applications, industrialization, anthropogenic activities, and climatic change produce emissions through which heavy metals are transported to ecological systems (biotic and abiotic components) (Sarkar, 2002; Singh et al., 2011). The causes of heavy metal stress are summarized in **Figure 1**. Heavy metals, including cadmium (Cd), arsenic (As), molybdenum (Mo), cobalt (Co), nickel (Ni), and copper (Cu), are transported to the environment through water and air. Some of these heavy metals are vital and required for the normal growth and production of plants, such as Fe, Zn, and Cu; however, these metals are required in trace amounts (Callender, 2005; Salomons et al., 2012; Alloway, 2013). Some heavy metals, even in trace amounts, cause toxicity in plant cells. Heavy metals accumulate in plant cells and cause heavy metal stress (Nagajyoti et al., 2010; Asati et al., 2016). Heavy metals are immobile and non-biodegradable, so they are toxic to the environment. When these heavy metals reach the soil through water, they are transported to plant cells through diffusion or endocytosis (Khan et al., 2015; Ali et al., 2020). Plants are sessile and face constant environmental stress, such as heavy metal stress, which is a kind of abiotic stress that harms plant productivity (Sharma and Agrawal, 2005; Ali et al., 2022). Heavy metal accumulation causes serious complications based on their subsequent accumulation throughout the food chain. Heavy metal stress adversely affects plant productivity by reducing enzymatic activities, soil biota, and metabolic reactions (Puschenreiter et al., 2005; Volpe et al., 2009; Sonone et al., 2020).

Interestingly, plants are natural bioaccumulators. They reduce the concentration of heavy metals in soil and accumulate them, although they do not require them (Smical et al., 2008). Plants have an intrinsic mechanism to tolerate stress, but only up to certain limits. After that, the plants show symptoms of toxicity, such as chlorosis, browning of the roots, growth reduction, and death. The response to heavy metals varies among several plant species (La Camera et al., 2004; Debnath et al., 2011; Zahra et al., 2022).

**Figure 2:**
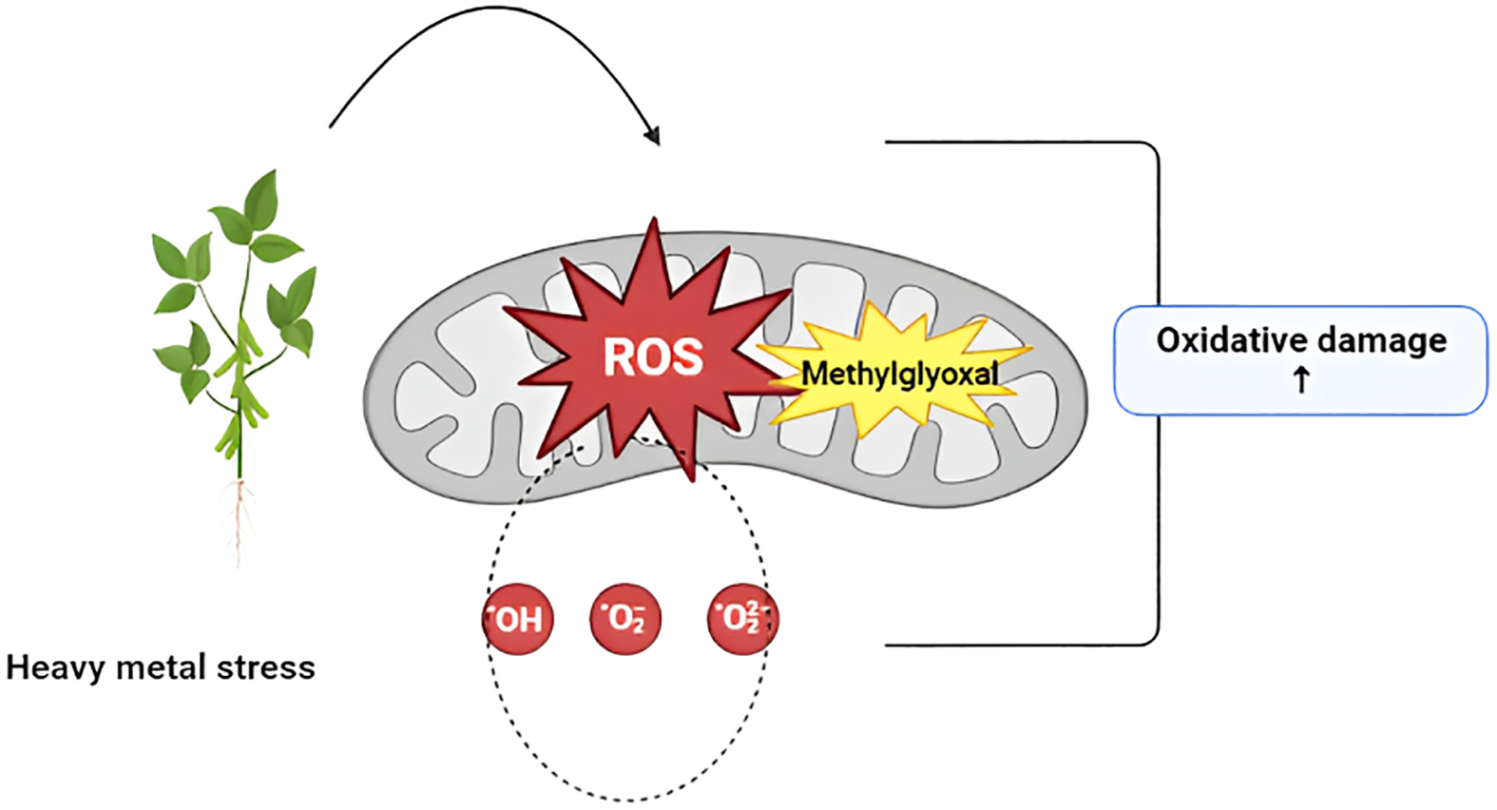
Production of oxidative damage in plants via ROS and methylglyoxal ROS (reactive oxygen species).

Whenever plants are exposed to heavy metals, they increase the production of free radicals such as reactive oxygen species (ROS), peroxide, superoxide, and singlet oxygen (Xie et al., 2019). These highly reactive diatomic molecules degrade biomolecules and cellular organelles. ROS results in oxidative damage to plant cells, as shown in **Figure 2** (Arora et al., 2002; Demidchik, 2015). Methylglyoxal (CH_3_CCHO) is an organic compound produced under heavy metal stress. When plants are subjected to heavy metals, the rapid accumulation of these substances results in ROS, causing oxidative stress that degrades carbohydrates (Hossain et al., 2012; Li, 2016; Yang et al., 2023). Carbohydrate degradation causes the production of a reduced derivative of pyruvic acid, resulting in cell toxicity. Methylglyoxal also inhibits the production of antioxidant enzymes, ultimately enhancing plant stress, as shown in **Figure 2** (Kaur et al., 2014; Mostofa et al., 2018).

As described earlier, plants are natural bioaccumulators. Therefore, whenever the amount of heavy metal reaches a specific limit, the first-line defense mechanism is related to stress (Kosakivska et al., 2021). The root exudates avoid heavy metal uptake (Dalvi and Bhalerao, 2013; Ghori et al., 2019). Metal ions are sequestered in different compartments to prevent metal interactions. The cell walls of the epidermis and mesophyll have negative charges. Therefore, metals can be accumulated by attracting negative charges, such as Cu. Heavy metals can also be stored as free ions in the vacuole of the cell (Krzesłowska, 2011). Sometimes, heavy metals form complex cellular proteins or salt complexes. Amino acids, organic acids, and antioxidants (enzymatic and non-enzymatic) are responsible for the tolerance mechanism of heavy metal stress (Zaets and Kozyrovska, 2012; Yu et al., 2019; Lei et al., 2021).

In response to continuous exposure to heavy metals, the avoidance and tolerance mechanisms become exhausted, and plants respond via the survival mechanism of action. The survival mechanism includes the process of detoxifying metals by producing stress-related molecules such as hormones, genes, and proteins (Egamberdieva et al., 2017).

**Figure 3:**
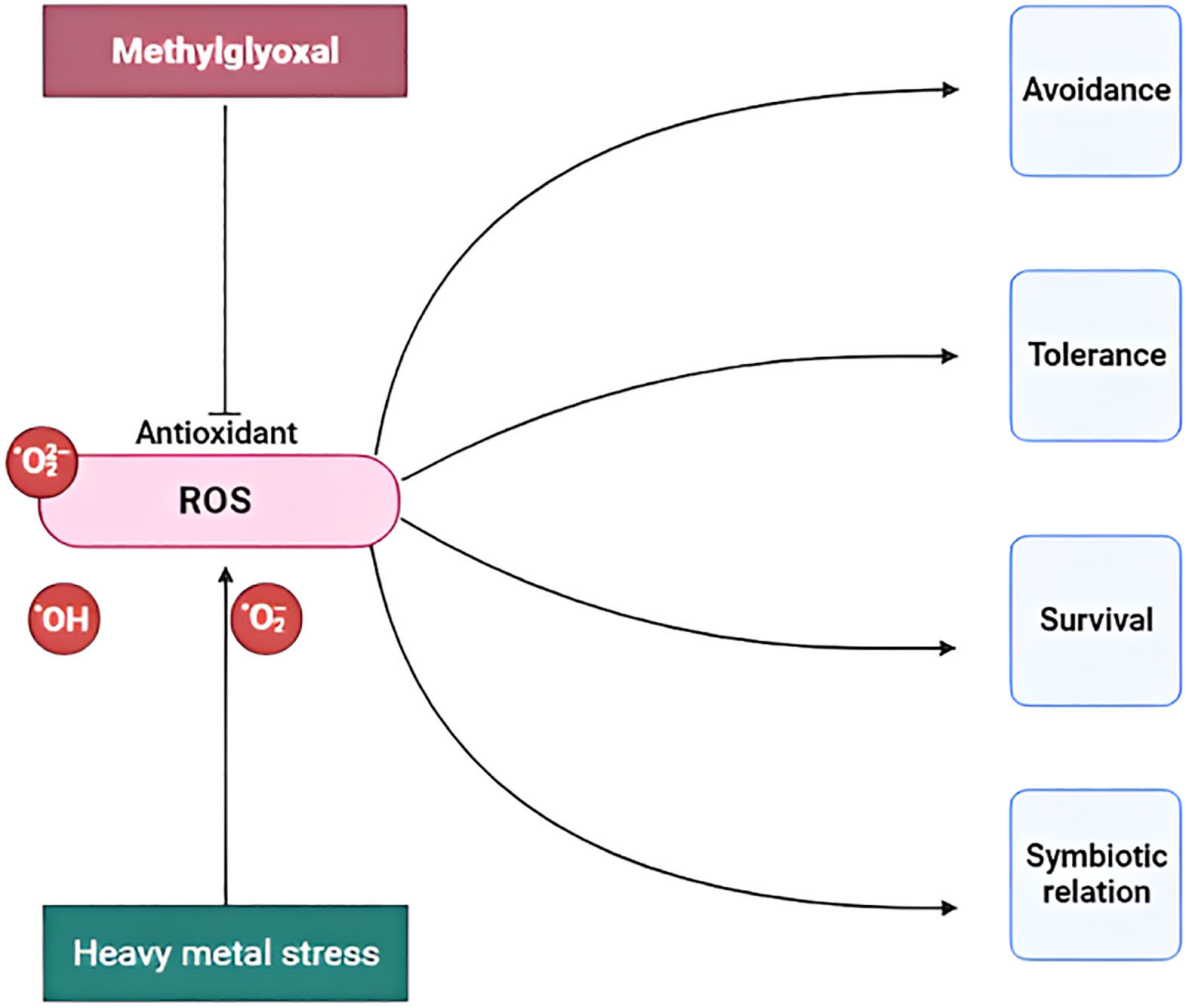
A holistic view of the metabolic reprogramming of plants with heavy metal stress ROS (reactive oxygen species).

Rhizospheric soil has different microbes, such as bacteria, nematodes, fungi, and viruses, which enhance stress tolerance. In addition, plants sometimes form biological relationships with rhizospheric microbes. This interaction is known as the plant–microbial interaction. This interaction enhances (1) the production of plant growth-promoting potential, stress-related phytohormones, and secondary metabolites; (2) the expression of genes and proteins; (3) the activation of antioxidant molecules; and (4) stress tolerance in plants. It regulates heavy metal stress via mitogen-activated protein kinases. It is a highly conserved signaling pathway connected with the phosphorylation and activation of hormones (Ali et al., 2022). The entire metabolic reprogramming process is provided in **Figure 3**.

Over time, plants adapt to heavy metal stress to endure it and maintain physiological growth, but only up to a limit. Plant species can tolerate different degrees of stress at different times, depending on the species, their habitats, and the duration of heavy metal stress. Plant cells may die if heavy metal stress becomes a long process (Bisht et al., 2023; Lu et al., 2023; Shakoor et al., 2023; Xu et al., 2023).

Soybean (*Glycine max*) is an important legume cultivated globally. It is an essential crop because of its nutritional value. It contains rich amounts of oil, proteins, fibers, minerals, and vitamins. Various studies have reported that abiotic stresses, such as heavy metal stress, severely threaten soybeans. The synergistic effect of heavy metal toxicity adversely influences soybean growth and metabolic processes.

Moreover, soybean is not tolerant of heavy metal stress; hence, metal-induced stress significantly affects its growth from germination to production. The toxicity also enters the food chain and affects human health. Metal accumulation in crop grains is considered of great concern, while phytobeneficial microbes are reported to have essential abilities for biosafety of crops grown in contaminated soil (Liu et al., 2021; Razi and Muneer, 2021). Soybean has a strong tendency and potential to build a symbiotic relationship with microbes (Chen et al., 2021; Zhou et al., 2023). The studies regarding mitigating the heavy metal stress via inoculation of the microbes are limited, and no single comprehensive study is present in soybean plant model. Reducing metal stress in soybean crops through a sustainable approach is extremely needed. Therefore, the present study was conducted to explore the role of microbes in mitigating heavy metal stress in model soybean plants. The present study reports for the very first time that phytohormone-producing microbes are coordinately involved in soybean growth and adaptation under heavy metal stress. Moreover, it explores the mutualistic association of the beneficial microbes in reducing heavy metal stress in the soybean plant without compromising its seeds or biochemical profile.

## Role of microbes in the alleviation of heavy metal stresses

Heavy metal stress is a kind of abiotic stress that negatively affects the productivity of various crops, such as tomato (Marques et al., 2023; Khan et al., 2023a), rice (Mao et al., 2022; Zhao et al., 2023), wheat (Din et al., 2023; Noor et al., 2023), millet (Revathy and Shanker, 2023; Khan et al., 2023b), maize (Hafeez et al., 2023; Tang et al., 2023), soybean (Lavado et al., 2001; Martin Molinero et al., 2023), and others. Heavy metal remediation is required for environmental protection and conservation. Numerous physicochemical and biological strategies have been used to remove heavy metals from the environment. Physicochemical procedures are quick, yet they are considered difficult due to their expense and technical complexity. They also have a negative impact on the physical, chemical, and biological aspects of the soil and contribute to secondary contamination. However, because biological remediation is a natural, eco-friendly, low-cost, and widely accepted technology, it is regarded as the most successful form of toxic metal elimination. One such method is the employment of plant growth-boosting bacteria for bioremediation of heavy metal-polluted soil, which is important in the context of biological degradation (Adesemoye and Kloepper, 2009; Chamkhi et al., 2021; Shaffique et al., 2022b).

**Figure 4:**
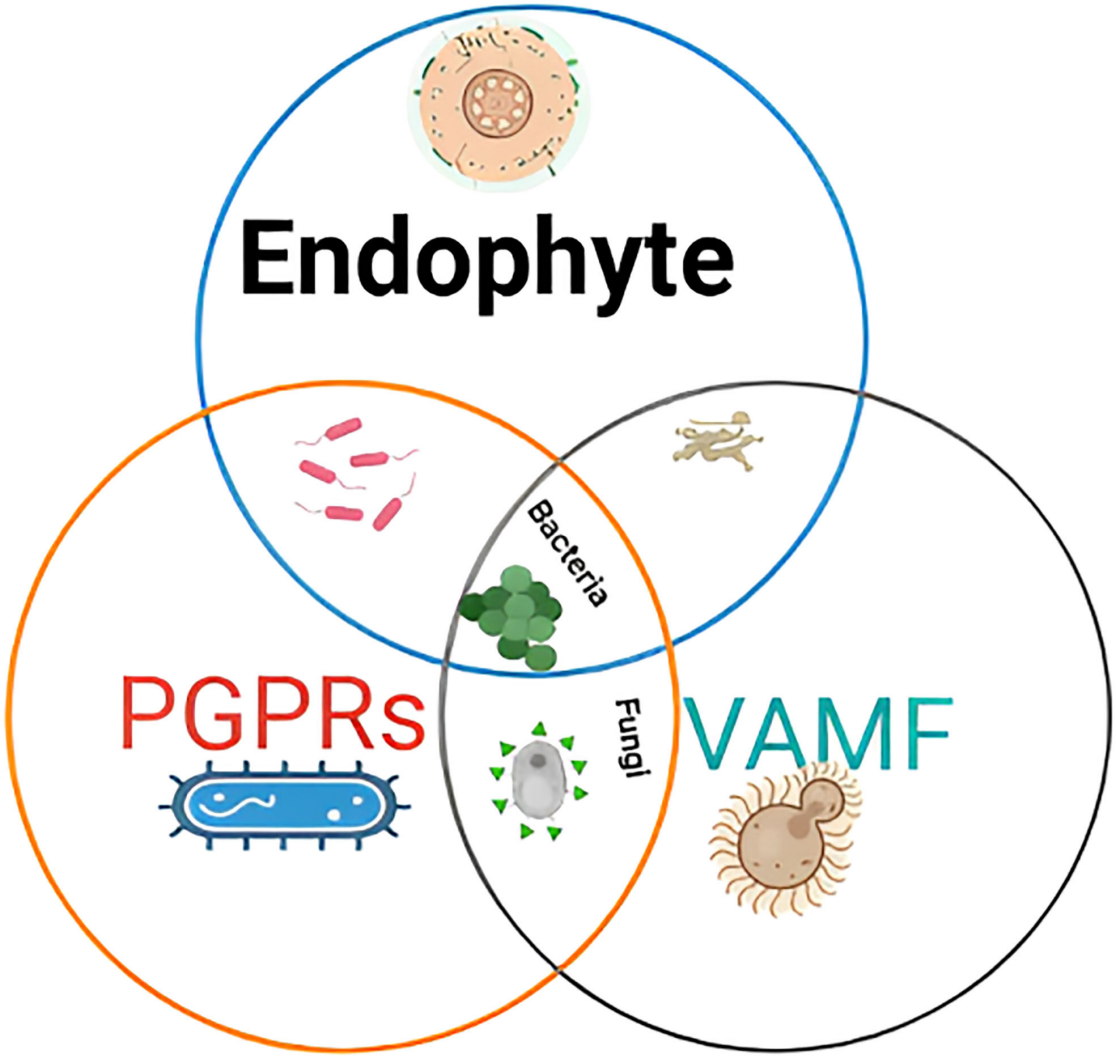
Microbes involved in the study for mitigation of heavy metal stress in soybean.

The inoculation of beneficial microbes mitigates heavy metal stress in plants. Microbes, such as bacteria, fungi, viruses, and nematodes, are used to minimize heavy metal stress (Ge et al., 2023; Narayanan and Ma, 2023; Rahman et al., 2023). Microbes have been acknowledged as an eco-friendly and alternative way to increase plant productivity and alleviate stress. However, the microbes potentially producing phytohormones are less evaluated for soybean (*G. max* L.). The current study focused on plant growth-promoting rhizobacteria (PGPR) and endophytes and their role in mitigating heavy metal stress, as shown in **Figure 4**. The interactive effect of microbes via the production of phytohormones and other cellular events on soybean growth and heavy metal stress tolerance is the least known.

## Plant–microbial interaction

Beneficial microbes (bacteria and fungi) are acknowledged for their stress tolerance and organic pollutant degradation. Plant–microbial interaction enhances plant growth even under abiotic stress (heavy metal stress). The microbes have the potential to integrate plant growth and metal accumulation via the production of essential metabolites such as ACC deaminase, organic acid, etc. (Prabhakaran et al., 2016; Tiwari and Lata, 2018). The metal tolerance mechanism is the induction of thiol compounds such as superoxide dismutase or metallothionein. Various studies reported that metal tolerance could be achieved in the plant via the inoculation of microbes mediated by the production of thiol compounds. The two pathways follow microbe-assisted heavy metal tolerance. One is the direct growth enhancement pathway, and another is the indirect growth enhancement microbial-assisted pathway. Microbes such as endophytes provide essential nutrients such as vitamins and minerals through a direct microbial-assisted pathway. This growth enhancement pathway works when the plant is under stress. In addition, the direct method also provides increased accumulation of phytohormones to enhance the biological process of the plant as well as increase stress tolerance in plants (Liu et al., 2023; Zhou et al., 2023). In direct microbial-assisted growth, the enhancement pathway is followed by the induction of systemic resistance (ISR). Various enzymes, such as proteases, glucanases, etc., assist the plant in the enhancement of resistance to metal stress (Gul et al., 2023; Nagrale et al., 2023).

**Figure 5:**
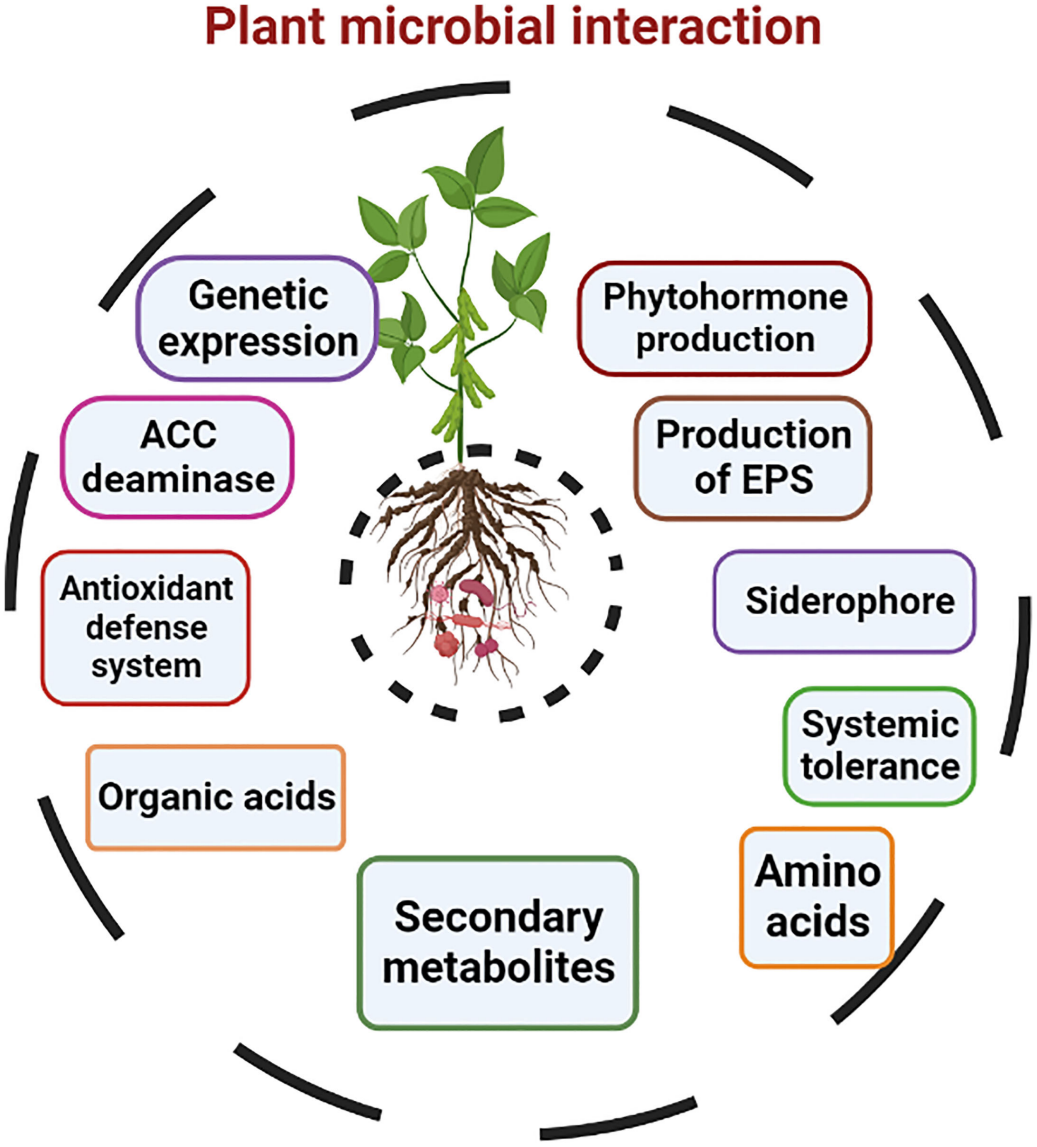
Plant growth-promoting mechanisms of microbes in metal stress tolerance.

Beneficial microbes integrate a complex symbiotic relationship with the plant and create a dynamic network of plant–microbial interaction. Plant–microbial interaction enhances heavy metal tolerance due to the production of a cascade of events, such as phytohormones, exopolysaccharides, and siderophores, organic acids, amino acids, secondary metabolites, and enhancing the antioxidant defense system, as shown in **Figure 5**. The expression of some genes facilitates this interaction. Moreover, plant–microbial interaction modulates a plant’s metabolic process, which helps enhance stress tolerance (Rajendran et al., 2003; Pishchik et al., 2016; Islam et al., 2021; Zhou et al., 2023).

## Endophytes

Endophytes are living microbes within plant cells that form a symbiotic relationship with plants via a cascade of events (Rajkumar et al., 2009). These events include the production of phytohormones, organic compounds, and genetic expression. These may be microbes or fungi (Adhikari et al., 2023; Ghorai, 2023; Li et al., 2023). They form healthy relationships with plants. The following are examples of inoculation of endophytes in model plant soybeans to enhance metal tolerance (Gupta et al., 2023; Rai et al., 2023).

A study was conducted in 2018 to determine the stress mitigation potential of two endophytic fungal strains, *Paecilomyces formosus* LHL10 and *Sphingomonas* sp. LK11, in soybeans under zinc and aluminum (Al) stress. Hormonal regulation, antioxidant capacities, and genetic expression were ruled out. The results suggest that inoculation of the endophytic strain significantly enhanced the production of the endogenous phytohormones abscisic acid, salicylic acid, and gibberellins. It also enhances the activity of antioxidant enzymes such as SOD, CAT, and APX. Furthermore, genetic expression revealed that it upregulates the overexpression of the Ariadne-like ubiquitin ligase gene, namely *GmARI1*, and downregulates the ATPase genes *GmHMA18*, *GmHMA13*, *GmHMA19*, and *GmPHA1*. The mechanism they followed for stress mitigation was inhibiting the uptake and reduction of oxidative stress by producing antioxidant molecules. The combined inoculation improved soybean’s metal toxicity and morphological features (Bilal et al., 2018).

Later in 2018, extending the work on endophytic fungi, Bilal et al. (2018) explored the mechanism of Cr tolerance in soybeans. The researchers estimated the presence of endogenous phytohormones and antioxidant molecules. The results revealed that inoculation of endophytic fungi significantly upregulated endogenous phytohormones, i.e., indole acetic acid, abscisic acid, and salicylic acid. The entophytic treatment mitigated Cr toxicity by enhancing the antioxidant defense system, such as catalase, superoxide dismutase, and peroxidase activities (Bilal et al., 2018).

In 2019, two entophytic strains, LH10 and LH6, were inoculated into soybeans to mitigate Ni, Cd, and Al. The results suggested that these strains upregulated the endogenous phytohormones (i.e., abscisic and jasmonic acids), reducing metal transportation and accumulating the genetic expression of *GmHMA13*, *GmHMA14*, and *GmHMA18*. The combined application enhanced the physio-morphological characteristics of the plants and reduced oxidative stress by activating antioxidant systems (e.g., GSH, SOD, APx, and CAT). (Bilal et al., 2020). Saqib et al., extending the work on *P. funiculosum* LHL06, estimated the quantitative measurement of phytohormones. Their results revealed that the inoculation of LH06 enhanced the production of the growth-promoting hormone gibberellins and indole acetic acid and lowered the levels of ABA and salicylic acid. The genetic expression analysis showed that inoculation significantly upregulated the FQR1-like 1 isoform X2 and peroxidase and downregulated the expression analysis of three genes: *GmHMA13*, *GmHMA14*, and *GmHMA19* (Bilal et al., 2019).

## Vesicular–arbuscular mycorrhiza fungi

Vesicular–arbuscular mycorrhiza fungi (VAMF) are a type of beneficial microorganism that forms the plant–microbial interaction by improving the soil’s plant nutrition and health (Xin et al., 2001; Chinnusamy et al., 2006).

AMF has the capacity to increase the defensive system of AMF-mediated plants, which can aid in the establishment of plants in soils contaminated with heavy metals and encourage growth and development. Food crops, fruits, vegetables, and soils may collect heavy metals, posing a number of health risks. Under aluminum stress, nitrogen absorption was positively improved by AMF interaction with wheat. According to Salehi et al. (2006), Tripathi (2014), and Chaturvedi and Malik (2019), plants grown in soils enriched with Cd and Zn exhibit significant suppression of shoot and root growth, leaf chlorosis, and even death. The effects of AMF on the accumulation of metals in plants have been the subject of numerous reports in the literature (Dar and Reshi, 2017). In the soybean model plant, only two studies are presented regarding heavy metal stress, as described below.

In 1990, American scientists explored heavy metal toxicity in soil. They mainly targeted Zn, Cd, and Mn metals. These researchers isolated some bacterial strains and some VAMF. The researchers inoculated VAMFs into soybeans under conditions of metal toxicity. Then, after harvesting, they accessed the mineral content and nitrogenase activity. The results revealed that the inoculation of VAMF enhanced metal stress tolerance and improved nitrogen and phosphorus content compared to the bacterial strain. The results also showed that it improved the plant’s biomass (Heggo et al., 1990).

## PGPR and their role in the enhancement of heavy metal stress tolerance in soybean

PGPR are beneficial microorganisms that promote the growth and development of plants (Ahemad, 2019; Barra Caracciolo and Terenzi, 2021). PGPR also alleviate stress by producing phytohormones, siderophores, exopolysaccharides, organic acids, secondary metabolites, antioxidant agents, and systemic tolerance in plants (Bhat et al., 2023; Jalal et al., 2023; Thomas and Archana, 2023).

In 2015, Kang conducted an experimental study in which soybeans were grown under Zn and Cu toxicity at a dosage of 100 μm. *E. asburiae* KE17 was inoculated into the plants. All physiological, biochemical, and endogenous phytohormone levels were determined. The results revealed that inoculation enhanced metabolic reprogramming by lowering the level of free amino acid leakage and the levels of ABA and SA that were higher in the stress condition. Microbial inoculation also enhances nutrient availability in soybean plants (Kang et al., 2015).

*Pseudocitrobacter anthropi* was inoculated in soybeans under Cr and As toxicity. The biochemical and hormonal assays were determined. The results suggested that the strain can tolerate metal stress up to 1,200 ppm, and it mitigates the stress by regulating endogenous phytohormones (IAA 59.02 µg/ml and GA 101.88 nM/ml) (Hussain et al., 2021). In 2019, Chinese scientists designed an experimental study in which they inoculated two microbes, *Trichoderma harzianum* L. and *Bacillus subtilis* L., along with bio-char, into soybeans under Cd stress. Three Cd concentrations were prepared (0, 10, and 30). The results revealed that combining bio-char with plant growth-promoting microbes significantly reduced metal uptake. Improve the growth and biomass of plants significantly (Haider et al., 2021). In another study, seven microbial strains, namely *Pseudomonas* sp. IMBG163, *Pseudomonas aureofaciens* IMBG164, *Paenibacillus* sp. IMBG156, *Klebsiella oxytoca* IMBG26, *Pantoea agglomerans* IMBG56, *Bradyrhizobium japonicum* IMBG172, and *Stenotrophomonas maltophilia* IMBG147, were inoculated into soybean under Zn, Cd, and Cu stress. Heavy metal stress was produced by polluting the soil at 30–300 mg concentrations. The results showed that the inoculation of microbes lowered the toxicity level 10 times lower than in the negative control group. The plants were protected from heavy metal stress due to the activation of phenolic compounds and glutathione-S-transfers. The results suggest that microbial inoculation significantly enhanced the antioxidant activity of 10 SMV in Cd soil (Zaets et al., 2010).

In 2017, two strains of plant growth-promoting rhizobacteria of *Penibacillus* sp. were selected for Cr-induced toxicity. The results showed that inoculating microbes significantly enhanced soybean growth under Cr stress. The expression of chromium reductases in soybeans enhanced the antioxidant capacity of the plant to tolerate stress (Wani et al., 2018). The studies mentioned above were about single inoculation, and the results suggested that it improved the performance of soybeans under metal stress. Later, an experimental study was designed in Argentina in 2019. The results suggested that double inoculation of two strains, *B. japonicum* E109 and *Azospirillum brasilense* Az39, enhanced stress tolerance under As stress in soybean plants. The inoculation followed the phytostablization method and enhanced the nitrogen content in soybean to alleviate the Cr stress (Armendariz et al., 2019).

The trend of utilizing microbes is becoming prevalent for alleviating environmental stress from plants due to their interactive effect. They provide substantial tolerance for overcoming hostile conditions (Shaffique et al., 2022a). After establishing the plant–microbial interaction, they modulate various plant biological processes, which augment stress mitigation. Establishing a plant–microbial interaction leads to beneficial changes in the host plant to mitigate heavy metal stress, which boosts overall plant fitness. The plant response to heavy metal stress induces the accumulation of phytohormones such as ABA, JA, SA, IAA, etc. and stress-responsive genes such as *GmHMA13*, *GmHMA18*, etc., as shown in **Table 1** (Rajendran et al., 2003; Bhandari and Bhatt, 2021; Islam et al., 2021). The increased accumulation of ABA causes restricted photosynthesis due to the closure of the stomata and, ultimately, growth restriction under metal stress. In comparison with plants that are inoculated with beneficial microbes, as shown in **Table 1**, they lower the accumulation of ABA and are reported to have the potential to mitigate heavy metal stress (Kumar and Verma, 2018; Jan et al., 2019).

**Table 1:** Microbes and their corresponding mechanisms of action by which microbes mitigate heavy metal stress in soybean.

Country, Year, Reference	Microbe	Strain	Mechanism of action	Metal stress
South Korea 2018 (Bilal et al., 2018)	Fungus Entophytic	LHL10 LK11	Down regulate *GmHMA13, GmHMA18, GmHMA19*, and *GmPHA1* Upregulate *GmARI1*, ABA,SA,GA↑	Al, Zn
South Korea 2019 (Bilal et al., 2020)	Entophytic fungi	LHL10 and LHL06	↑ABA,JA Inhibit metal translocation Down regulation of *GmHMA13*, *GmHMA14*, and *GmHMA18* ↑Antioxidant	Ni, Cd, and Al
Pakistan 2021 (Hussain et al., 2021)	PGPRs	C18	↑Antioxidant ↑IAA,GA	Cr Ar
South Korea 2018 (Bilal et al., 2018)	Entophytic fungi	Lk11	↑IAA ↑ABA ↑SA ↑Antioxidant defense system	Cr
USA 1990 (Heggo et al., 1990)	vesicular–arbuscular mycorrhiza	NA	↑P 20%–87% ↑N ↑Biomass	Zn Cd Mn
South Korea 2019 (Bilal et al., 2019)	Endophytic fungi	LH06	↓ *GmHMA13*, *GmHMA14*, *GmHMA19*) and *GmMATE1* ↑GA,IAA ↓ABA,SA	Ni Cu Pb Cr and Al
China 2021 (Haider et al., 2021)	PGPRs	*Trichoderma harzianum L.* and *Bacillus subtilis L*	↓Cd bioavailability ↑Growth ↑Photosynthesis	Cd
South Korea 2015 (Kang et al., 2015)	PGPRs	*Enterobacter asburiae* KE17	↑Phytohormone ↑Antioxidant	Cu Zinc
Ukraine 2010 (Zaets et al., 2010)	PGPRs	IMBG 164 IMBG 163 IMBG 156 IMBG 147 IMBG 172	↑Carbonylated proteins ↑GPX ↑GST	Cu Zinc Cadmium
Iran 2017 (Wani et al., 2018)	PGPRs	*Penibacillus* MA12 MA11	Chromium reductases ↑Antioxidant	Cr
Argentine 2019 (Armendariz et al., 2019)	PGPRs	E109 AZ39	↑N content ↑ Phytostablization	Ar

The ↑and ↓represent the increase and decrease of the specific response.

This study suggests an increasing interest in the microbial field in mitigating heavy metal stress in plants. The mitigation of abiotic stress by microbes was first reported in 1990, and later there was a research gap. Only after 2000, especially from 2015 onward, was there an increasing interest in this research topic. Despite several studies evaluating heavy metal stress, they lack essential crop soybeans, so the most interest was seen. Now, more researchers around the world are expected to invest more effort in the field of mitigation of heavy metal stress by microbes.

## Conclusion and future prospective

**Figure 6:**
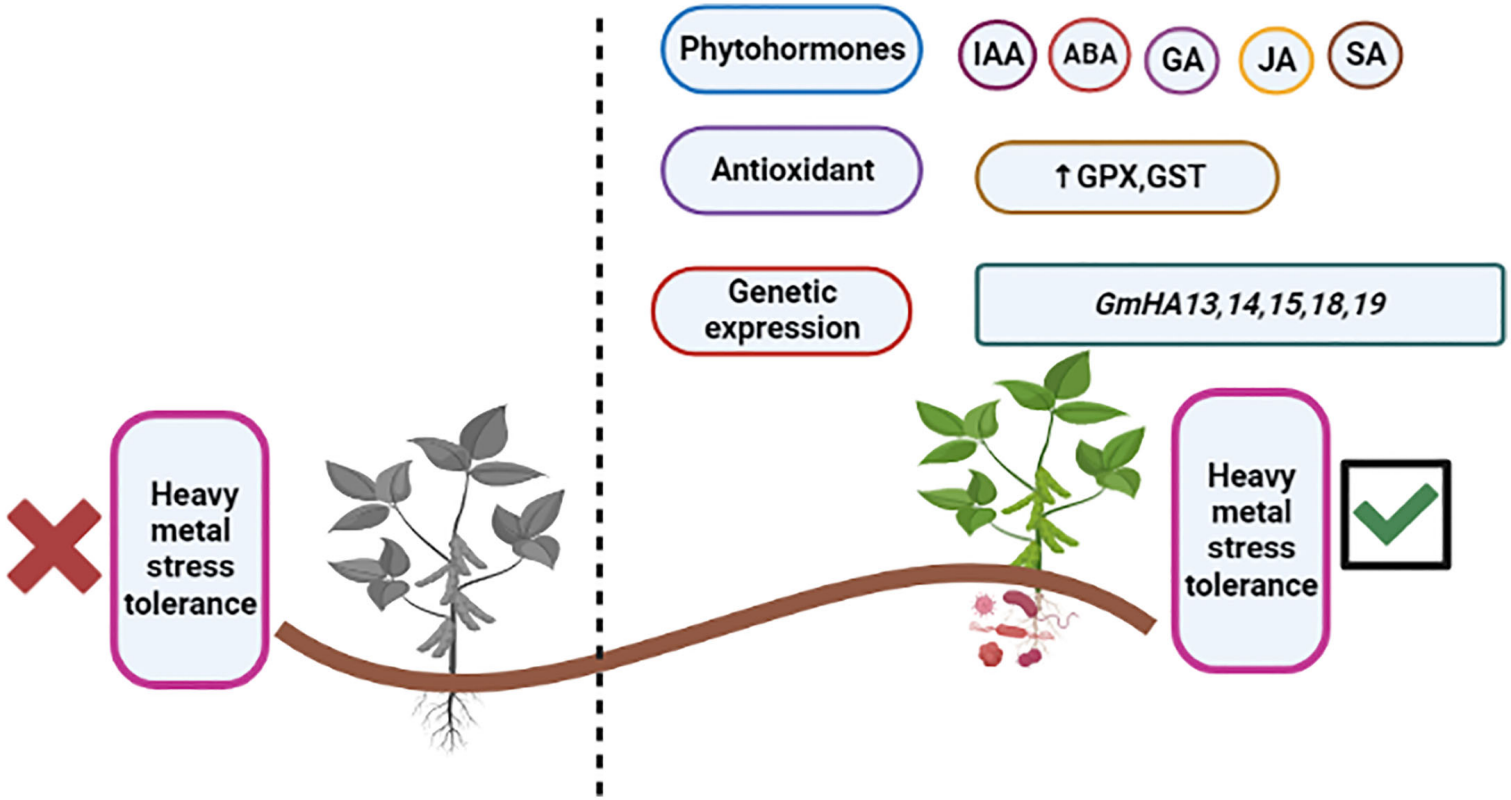
Mitigation of heavy metal stress via plant–microbial interaction.

Soybean is an important crop that is continuously threatened by heavy metal stress. Although many databases have been presented on heavy metal tolerance by inoculating microbes, only limited studies exist on the soybean model plant and heavy metal tolerance. This study concluded that microbes could enhance soybean growth under heavy metal stress. Microbial inoculation, such as plant growth-promoting rhizobacteria, VAMF, and endophytes, significantly improves heavy metal stress in many ways. These include the production of phytohormones, genetic expression, nitrogen fixation, phytostabilization increase, and antioxidant stress enhancement, as shown in **Figure 6**. In conclusion, we can say that phytohormones and phytohormone-producing microbes upregulated production of the reactive oxygen species production and then triggered the attenuation of heavy metal stress via antioxidant pathways such as the production of GSH. Phytohormones have an important role in the mitigation of heavy metal stress. Among them, ABA, JA, SA, and ethylene have a synergistic effect on stress tolerance. Microbial inoculation opens a new door for researchers in the field of enhancing heavy metal stress tolerance. Finally, the study suggested that compatible microbial isolates could be an eco-friendly and vital strategy for developing soybean and metal tolerance in multi-metal-contaminated soil and a sustainable approach to agronomy. The present study suggests further studies investigating the synergistic interaction of reported microbes with other beneficial microbes in the rhizosphere as well as large-scale *in situ* studies for bioremediation of multi-metal-contaminated soil under hostile environmental conditions.

## Author contributions

SS wrote and conceptualized the original draft. MI and SH did the critical review editing. E-HK, MK, and S-MK did the formatting. I-JL validated and supervised the manuscript and provided the funding. All authors contributed to the article and approved the submitted version.
